# Oxidation State, A Long-Standing Issue

**DOI:** 10.1002/anie.201407561

**Published:** 2015-03-10

**Authors:** Pavel Karen

**Affiliations:** Department of Chemistry, University of OsloP.O. Box 1033 Blindern, 0315 Oslo (Norway)

**Keywords:** bond graphs, bond order, bond valence, Lewis formulas, oxidation state

## 1. Introduction

The oxidation state is the simplest attribute of an element in a compound. It is taught early in the chemistry curriculum as a convenient electron-counting scheme for redox reactions. Its applications range from descriptive chemistry of elements to nomenclature and electrochemistry, or as an independent variable in plots and databases of bonded-atom properties (such as radius, bond-valence parameter, standard reduction potentials, spectral parameters, or spin).

The history of the oxidation state goes back about 200 years when it described the stepwise increase in the amount of oxygen bound by elements that form more than one oxide. In his 1835 textbook *Unorganische Chemie*,[1] Wöhler speaks of such an “oxydationsstufe” (an older German spelling for oxidation grade). This expression remains in use for oxidation state in several languages. The equivalent term oxidation number is also common; in English this refers more to redox balancing than to the chemical systematics of an element.[2]

Under the entry for oxidation number, the IUPAC “Gold Book”[3] gives a defining algorithm for the oxidation state of a central atom as the charge it obtains after removal of its ligands along with the shared electron pairs. The entry for oxidation state in Ref. [3] complements this with a set of charge-balance rules and of postulated oxidation states for oxygen and hydrogen with exceptions. Details vary from textbook to textbook. Some list the rules according to decreasing priority to avoid the explicit exceptions; here is an example:[4]

1. Atoms in an element have oxidation state 0.
2. The sum of the oxidation states for atoms in a compound is 0.
3. Fluorine in compounds has the oxidation state −1.
4. Alkaline metals in compounds have the oxidation state +1, alkaline-earth metals +2.
5. Hydrogen in compounds has the oxidation state +1.
6. Oxygen in compounds has the oxidation state −2.

In recent debates, Steinborn[5] and Loock[6] advocate Pauling’s[7] approach of assigning shared electron pairs to the more electronegative atom. Jensen[8] elaborates on some of the points considered by Loock. Smith[9] and Parkin[10] address the oxidation state in the context of related terms. Calzaferri[11] as well as Linford and co-workers[12] make suggestions on the oxidation state of organic compounds. Jansen and Wedig[13] point out the heuristic nature of the oxidation state and require that “*concepts need to be defined as precisely as possible, and these definitions must always be kept in mind during applications*”.

IUPAC also realized the need to approach a connotative definition of the oxidation state. In 2009, a project was initiated “Toward Comprehensive Definition of Oxidation State”, led by the author of this Essay, and its results have recently been published in an extensive Technical Report.[14] We started with a generic definition of oxidation state in terms broad enough to ensure validity. Then we refined those terms to obtain typical values by algorithms tailored for Lewis, summary, and bond-graph formulas.

## 2.Generic Definition

The oxidation state is the atom’s charge after ionic approximation of its bonds. The terms to be clarified are the “atom’s charge”, “its bonds”, and the “ionic approximation”.

The atom’s charge is the usual count of valence electrons relative to the free atom. The oxidation state is a quantitative concept that operates on integer values of counted electrons. This may require idealizing visual representations or rounding off numerical results.

Approximating all bonds to be ionic may lead to unusual results. If the N=N bond in N_2_O were extrapolated to be ionic, the central nitrogen atom would have an oxidation state of +5 and the terminal one −3. To obtain less extreme values, bonds between atoms of the same element should be divided equally upon ionic approximation.

Several criteria were considered for the ionic approximation: 1) Extrapolation of the bond’s polarity; a) from the electronegativity difference, b) from the dipole moment, c) from quantum-chemical calculations of charges. 2) Assignment of electrons according to the atom’s contribution to the molecular orbital (MO).

**Figure 1:**
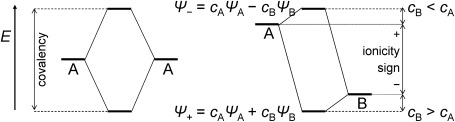
The essence of the adopted ionic approximation based on the contribution to the bonding MO. The mixing coefficients *c*_A_ and *c*_B_ refer to the atomic-orbital wavefunctions *ψ*_A_ and *ψ*_B_ in an MO-LCAO approach (LCAO=linear combination of atomic orbitals).

As discussed in Appendix B of Ref. [14], most electronegativity scales depend on the atom’s bonding state, which makes the assignment of the oxidation state a somewhat circular argument. Some scales lead to unusual oxidation states, such as −6 for platinum in PtH_4_^2−^ with Pauling or Mulliken scales. Appendix E of Ref. [14] shows that a Lewis-basic atom with an electronegativity lower than its Lewis-acidic bond partner would lose the often weak and long bond upon ionic approximation of their adduct, thereby yielding an unusual oxidation state. Appendix A of Ref. [14] points out that dipole moments of molecules such as CO and NO, which are oriented with their positive end towards oxygen,[15–17] would lead to abnormal oxidation states. Appendix C of Ref. [14] illustrates the variety of calculated quantum-chemical atomic charges. This leaves the atom’s contribution to the bonding MO, the atomic-orbital energy, as the criterion for ionic approximation (Figure 1).

Figure 1 implies that while AA bonds are divided equally, in an AB compound the atom contributing more to the bonding molecular orbital receives negative charge under ionic approximation of the bond. Ref. [14a] emphasizes that the said contribution does not concern the actual origin of the bond’s electrons upon its formation, only their final allegiance. Figure 1 is not an instruction to use the mixing coefficients; it merely illustrates a concept. The same ionic approximation is obtained when the more heuristic orbital energies are considered.

## 3. Simple Estimate of Ionic Approximation

**Figure 2:**
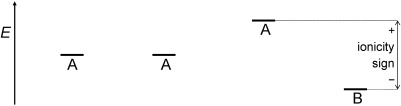
The ionic approximation according to the relative energies of the free-atom valence orbitals, conveniently derived from Allen’s electronegativities.

Should complicated MO schemes make the above criterion impractical, the ionic approximation can be estimated from electronegativities. Of several scales discussed in Appendix B of Ref. [14], only the Allen electronegativity is truly independent of the oxidation state, as it relates to the average valence-electron energy of the *free* atom.[18–20] Such an ionic approximation is obtained when the bonds implied in Figure 1 are abstracted away (Figure 2).

The electronegativity criterion for the ionic approximation carries an exception if the more electronegative atom is reversibly bonded as a Lewis acid (a so called Z-ligand, Appendix E of Ref. [14]): Its acceptor orbital is high, and the less-electronegative Lewis-base donor atom retains the electrons because of its larger contribution to the bonding MO. An allegiance criterion by Haaland[21] identifies such an adduct: Applied to ionic approximation, one asks where the bonding electrons go when the bond is split thermally. If the split is heterolytic, the ionic approximation follows the electrons; if homolytic, electronegativity applies. Table 1 lists the Allen scale.

**Table 1:** Allen electronegativities^[18–20]^ (in Pauling units).

H 2.300							He 4.16		
									
Li 0.912	Be 1.576	B 2.051	C 2.544	N 3.066	O 3.610	F 4.193	Ne 4.787		
									
Na 0.912	Mg 1.293	Al 1.613	Si 1.916	P 2.253	S 2.589	Cl 2.869	Ar 3.242		
									
K 0.734	Ca 1.034	Ga 1.756	Ge 1.994	As 2.211	Se 2.424	Br 2.685	Kr 2.966		
									
Rb 0.706	Sr 0.963	In 1.656	Sn 1.834	Sb 1.984	Te 2.158	I 2.359	Xe 2.582		
									
Cs 0.659	Ba 0.881	Tl 1.789	Pb 1.854	Bi 2.01	Po 2.19	At 2.39	Rn 2.60		
									
Sc 1.19	Ti 1.38	V 1.53	Cr 1.65	Mn 1.75	Fe 1.80	Co 1.84	Ni 1.88	Cu 1.85	Zn 1.59
									
Y 1.12	Zr 1.32	Nb 1.41	Mo 1.47	Tc 1.51	Ru 1.54	Rh 1.56	Pd 1.58	Ag 1.87	Cd 1.52
									
Lu^[a]^ 1.09	Hf 1.16	Ta 1.34	W 1.47	Re 1.60	Os 1.65	Ir 1.68	Pt 1.72	Au 1.92	Hg 1.76

[a] The variation across the lanthanoid series has not been evaluated.

## 4. Algorithm for Summary Formulas

The octet rule[22] concerns the most electronegative atoms in the periodic system. On a sufficiently simple summary formula involving such atoms, it alone dictates the oxidation states. The algorithm is named DIA (direct ionic approximation) in Ref. [14]: Atoms are assigned octets according to their decreasing electronegativity until all the available valence electrons are used up. The atom charges then represent the oxidation states.

**Figure 3:**
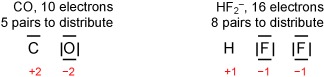
Oxidation states (in red) in CO and HF_2_^−^ from DIA (direct ionic approximation) performed on a summary formula by distributing valence electrons (here drawn in pairs) into octets according to decreasing electronegativity.

Typical DIA-friendly species are homoleptic binaries of at least one sp element (Figure 3): CO, HF_2_^−^, NO_3_^−^, NO_2_, NH_4_^+^, CrO_4_^2−^, BF_4_^−^, SF_6_, SnCl_6_^2−^, CuCl_4_^2−^, RuO_4_, AuI_4_^−^…; or solids with a homoleptic periodic bonding unit: KBr, SiC, AlCl_3_, SnCl_2_, etc. DIA of compounds of three or more elements may become ambiguous, with the limitations discussed in Appendix D of Ref. [14].

## 5. Algorithm of Assigning Bonds

**Figure 4:**
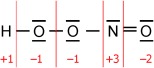
Oxidation states (in red) in peroxynitrous acid, obtained by assigning bonds to more electronegative partners on Lewis formula with all valence-electron pairs drawn (dashes).

These algorithms work on Lewis formulas that display all the valence electrons: Bonds are assigned to the more negative bond partner identified by ionic approximation. The resulting atom charges then represent the oxidation state (Figure 4). As only homonuclear bonds are divided (equally), the correct bond multiplicity is essential only between those pairs of atoms of the same element that appear asymmetrical within the segment of the pair’s bonds, including the sign of their ionic approximation: Whereas the OO bond order in Figure 4 would not matter so long as the -OO- segment were kept symmetrical, the NN bond order in an N_2_O Lewis formula always matters.

**Figure 5:**
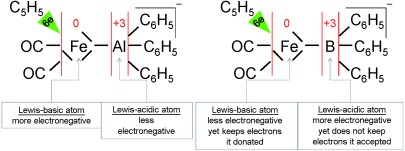
Oxidation states (in red) by assigning a metal–metal bond according to the atoms’ contributions to the bonding MO. An assignment according to the electronegativity needs to invoke the caveat of a Lewis-basic atom with electronegativity lower than the Lewis-acidic one (in the formula on the right).

An example of the exception to the rule of ionic approximation according to electronegativity is [(C_5_H_5_)(CO)_2_Fe=B(C_6_H_5_)_3_][23] on the right-hand side of Figure 5. Despite the higher electronegativity of B, the Lewis-basic Fe atom keeps the electrons it donated to bond triphenylborane. When B is replaced by Al[24] (Figure 5 left), the same principle applies, now in line with the Fe and Al electronegativities. The weak donor–acceptor bonds in these two adducts are the telltale sign of the reversibility criterion of Haaland,[21] suggested in Ref. [14] to identify cases of electron allegiance against electronegativity such as the one on the right-hand side of Figure 5.

## 6. Algorithm of Summing Bond Orders

**Figure 6:**
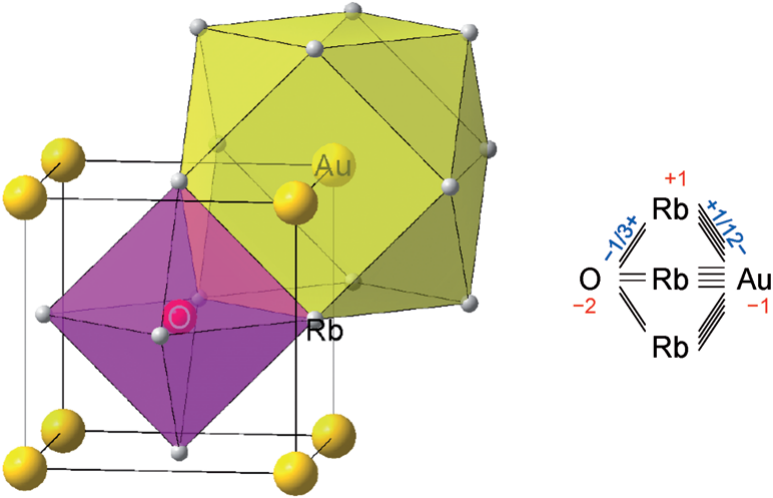
The unit cell and coordination polyhedra of the AuORb_3_ perovskite with its bond graph of ideal bond orders (values in blue) obtained from the 8−*N* rule for O, the 8+*N* rule for Rb, and the 12−*N* rule for Au. The bond orders are shown with signs, which sum up at each atom to yield that atom’s oxidation state (in red).

This algorithm is tailored to bond graphs. A bond graph represents the infinite periodic network of an extended solid.[25,26] It is constructed on a stoichiometric formula of the network’s repetitive unit, with atom symbols distributed such that a straight line is drawn for each instance of an atom’s bonding connectivity. Each line carries its own specific bond order. To obtain the oxidation state, a sum is calculated at each atom, of the orders of its bonds weighted by their ionic sign at that atom. Such an “ionized bond order sum”, *iBOS*, then equals the atom’s oxidation state. Figure 6 explains this on the AuORb_3_ perovskite-type structure[27] with bond orders according to the 8+*N* rule at Rb, 8−*N* rule at O, and the 12−*N* rule at Au.

The 8+*N* rule: An electropositive sp atom with *N* valence electrons forms *N* two-electron bonds with atoms of higher electronegativity. The rule concerns alkali metals and alkaline-earth metals. The 8 in its name symbolizes the preceding noble-gas shell.

The 8−*N* rule: An electronegative sp atom with *N* valence electrons tends to form 8−*N* but not more than four two-electron bonds with atoms of equal or lower electronegativity. As an example, phosphorus with 5 valence electrons forms 3 two-electron bonds in the P_4_ tetrahedron, and nitrogen does the same in N_2_. In heteroatomic molecules, the 8−*N* rule is enforced by higher electronegativity. For example: In sulfur fluorides, the 8−*N* rule concerns fluorine. Bonds in SF_2_, SF_4_, and SF_6_ have all approximately the length of a single bond.[28] In the series BF, CO, and N_2_, the full triple bond suggested by the octet rule only occurs in N_2_, whereas O and F force the bond order towards 2 and 1, respectively.[29] The 8−*N* rule is not the same as the octet rule, as each can be violated independently: The Lewis formula of N_2_O can be drawn as |N]N-O|, which has octets yet violates the 8−*N* rule on oxygen. Hydrogen obeys an analogous 2−*N* rule.

The 12−*N* rule: An element having close to 12 dsp electrons in its outermost shells tends to lose those that exceed 12 or to gain in bonds those less than 12. While the first tendency (to form s^2^ cations) is magnified by other trends in the main groups of the periodic system, the second (to form s^2^ anions) is specific to Pt and Au, which in such compounds are called relativistic chalcogens and halogens, respectively.[30]

**Figure 7:**
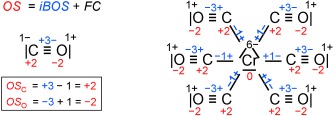
Oxidation state (in red) in carbon monoxide (left) and chromium hexacarbonyl (right) calculated from Lewis formulas of idealized bond orders (in blue) featuring nonzero formal charges (in black).

Besides bond graphs, the algorithm works on Lewis formulas that display bond orders. It works directly if the formula does not carry formal charges. If it does, the atom’s formal charge *FC* is added to the atom’s positive or negative sum of bond orders *iBOS* to yield its oxidation state [Eq. (1)].[14b] This relationship is illustrated for CO and [Cr(CO)_6_] in Figure 7.

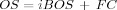
(1)

The bond orders in extended solids are not always obvious and may have to be estimated from bond lengths. This is done by converting each bond length into the so-called bond valence, which is a value entirely equivalent to the bond order in terms of two-electron bonds in molecules. The origins of the bond-valence approach—one[31] ionic and one[32] covalent—are associated with Linus Pauling. In Ref. [32], an expression is given that morphed into the current relation for bond valence versus bond length [Eq. (2)].

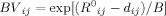
(2)

In this expression, *BV_ij_* and *d_ij_* are the respective bond valence and distance of the atoms *i* and *j*, *R*^0^_*ij*_ is the single-bond length between them, and *B* is a variable parameter often fixed to 0.37. Although for the best accuracy *R*^0^_*ij*_ is a function of the coordination number and oxidation state of the “cation” for a given “anion” (fitted[33,34] to a set of such structures), a general approach[35] lists two parameters for each atom, related to the size and electronegativity, from which *R*^0^_*ij*_ is calculated for any atom pair *i* and *j*. As the oxidation state operates on integer electrons, a round off is required on the obtained bond valences/orders or on their ionized sums at an atom.

**Figure 8:**
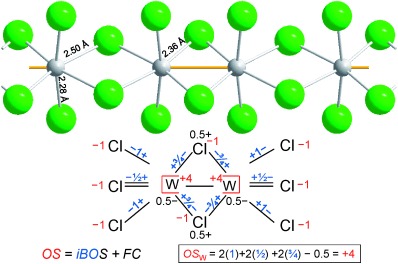
Top: A segment of the infinite WCl_4_ chain with alternate W=W bonds. Bottom: Bond graph of the chain with idealized bond orders (in blue, rounded off bond-length to bond-valence calculations), which sum at each atom with the formal charges (in black) to the oxidation state of that atom (in red).

We will now analyze WCl_4_ in Figure 8. The bond graph of its infinite chain has a W-W group and eight Cl atoms, with each W coordinating six Cl atoms by two single, two 3/4, and two 1/2 bonds, as estimated from experimental bond lengths[36] in Ref. [14c]. Participation of more than one p orbital at the Cl bridge above and below W-W makes its bond-order sum (1.5 at Cl) exceed the 8−*N* rule. A chain edge resembles locally a molecule, and such a Cl atom in a Lewis formula would carry a formal charge of 0.5+, compensated by 0.25− at each of the two W atoms it bridges. This feature can be seen in the bond graph in Figure 8.

**Figure 9:**
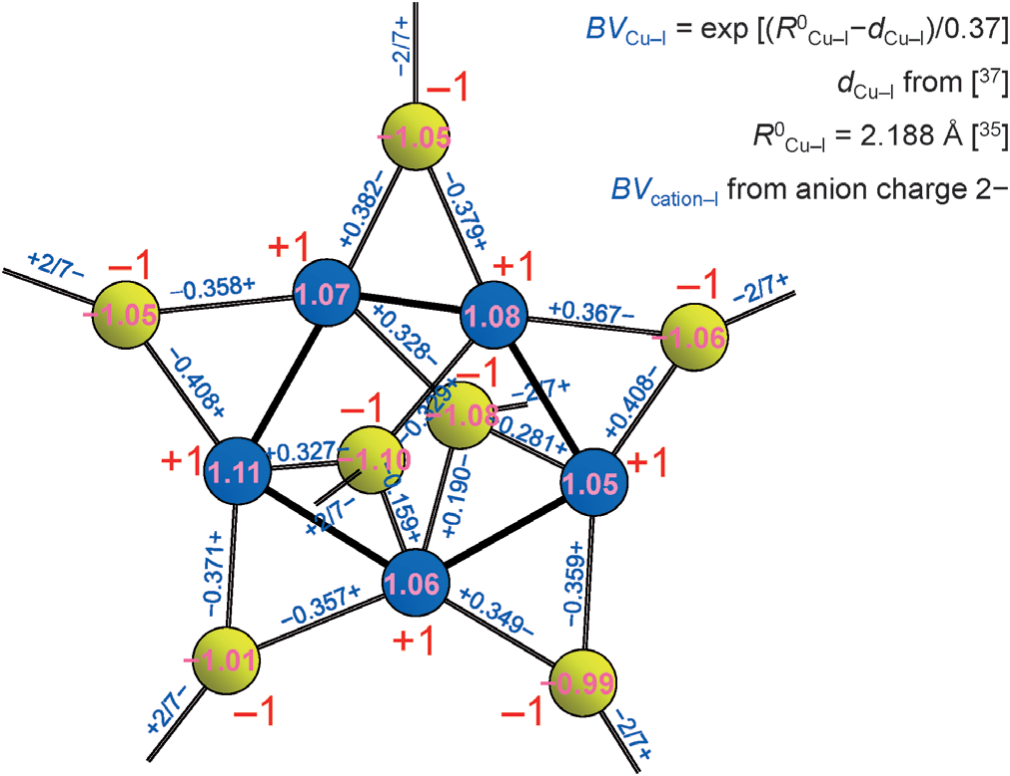
Bond valences (in blue) in Cu_5_I_7_^2−^ and the sums of the ionized bond valence at the atoms (in pink) that yield the oxidation states (in red) upon rounding off. Blue sphere Cu, yellow sphere I.

The bond-valence approach to bond orders can also be used for finite species. An example is Cu_5_I_7_^2−^ [37] in Figure 9. One of the five Cu atoms bonds to four iodine atoms, while the remaining four Cu atoms bond three. Two of the seven iodine atoms bond to three Cu atoms and the remaining five only to two. The oxidation state is evaluated as a round off value of the positive and negative sums of bond valences calculated with Equation (2)] from bond distances *d*_CuI_ in Ref. [37] and *R*^0^_CuI_=2.188 Å obtained from parameters in Ref. [35]. As the Cu=Cu bonds are not approximated to be ionic, only the CuI bonds are relevant besides the 2/7 bonds from each iodine atom to the cations omitted in Figure 9, which yields ionized bond valence sums of −1.05(4) per iodine and +1.07(2) per Cu atom. A round off yields a single oxidation state for each element in a nice demonstration of the Pauling’s[31] parsimony rule.

An example of an sp molecule shows another kind of bonding compromise: (C_6_H_5_)_3_P]C]P(C_6_H_5_)_3_. This extreme Lewis formula emphasizes the high order of the phosphorus-to-carbon bond because of the 8−*N* rule working for the more electronegative carbon atom. A bond order of 1.9 is calculated as above from the distance[38,39] of 1.63 Å. As the PCP angle is not 180° but only 134°,[39] the 8−*N* rule is somewhat violated due to the only small difference in the electronegativity of P and C, and it is clear that this bond has a strong ionic contribution from the formal charges 1+, 2−, and 1+ that would appear on the PCP segment if σ bonded. As these charges comply with the electronegativity, the bond strengthens and becomes a[40] “sort of double bond”. When this ionocovalent interaction is drawn with two full dashes as above, the formula loses its formal charges, and the oxidation states equal directly to the sums of the ionized bond order: −4 at the carbon and +5 at the phosphorus atom. That makes sense redox-wise within the molecule[41] as well as in its full[42,43] synthesis. Given the bond strength, the Haaland criterion is unlikely to apply in this case.

**Figure 10:**
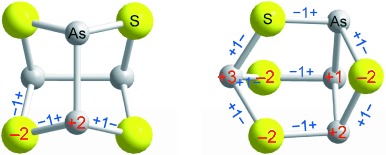
Oxidation states (in red) obtained by summing the bond orders (in blue) in two σ-bonded As_4_S_4_ clusters that maintain the 8−*N* rule.

An example of an sp cluster is As_4_S_4_. It occurs as two different molecules, where both elements maintain the 8−*N* rule (an electron-precise cluster). This information is sufficient to obtain their oxidation states by sums of the ionized bond orders (Figure 10).

**Figure 11:**
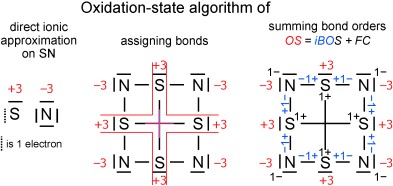
Oxidation states evaluated on formulas approximating S_4_N_4_: a periodic unit of a ring (left; neglecting the weak bonds among the sulfur atoms in the real compound) and a Lewis formula of octets (middle and right; approximating the tetrahedral sulfur cluster with two S=S bonds).

A cluster where the 8−*N* rule is weakened due to steric compromise is S_4_N_4_. It is the same as on the left side of Figure 10, except that N replaces S while S replaces As. Neither atom complies with the 8−*N* rule, which would require formation of three short two-electron bonds from the small N to the bulky S atom. As the bond lengths and angles in solid S_4_N_4_[44] are irregular, data for gaseous S_4_N_4_[45] are considered here: The molecule has a sulfur tetrahedron with a 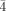 point symmetry and bond lengths of 2.666 and 2.725 Å, far longer than a single bond of 2.055 Å.[46] One approach to sensible oxidation states is to neglect these weak S-S interactions and consider S_4_N_4_ a cyclic tetramer with an SN summary formula, to which DIA applies to yield +3 and −3 for the oxidation states. Bond-based algorithms give the same result (Figure 11) on a symmetrical Lewis formula with 22 electron pairs, by considering the SNS bond angle of 105.3(7)° is tetrahedral and complemented by two lone pairs of electrons at N, with each S atom forming one single bond to another S atom (with a 1− formal charge at N and 1+ charge at S). The reality is somewhere in between these two simplifications: The S=N bond valence calculated with parameters from Ref. [35] with a S=N bond length of 1.623(4) Å[45] is 1.40(2)—more than the single bond of the Lewis formula but not quite the 1.50 required by the 8−*N* rule. The similarly calculated sum of the homonuclear bond valence at each S atom of the tetrahedron is about 0.5, half of the 1.00 generated by the single bond in the Lewis formula.

In general, the bonding in nonmetallic sp binary compounds C_*c*_A_*a*_ is rationalized with the Zintl concept[47] and its formalization with the generalized 8−*N* rule[48,49] [Eq. (3)]. In this relation, *VEC*_A_ is the valence-electron count per “anion” A, *CC* is the number of electrons per “cation” C that form C=C bonds or are localized at the cation as lone pairs, and *AA* is the number of electrons per A that form A=A bonds. The *VEC*_A_ value lends itself to a verbal approach to the rule: Whereas electrons in excess of 8 remain at the less-electronegative atom as bonds or lone pairs, electrons short of 8 are gained by forming bonds between the more electronegative atoms.

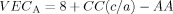
(3)

For our S_4_N_4_ example above, we obtain *VEC*_A_=11. The three electrons in excess of eight remain at the S atom as one S=S bond and one lone pair per S atom in our Lewis formula; in reality this is somewhat violated, since electronegativity makes sulfur enforce the 8−*N* rule almost as strongly as nitrogen.

**Figure 12:**
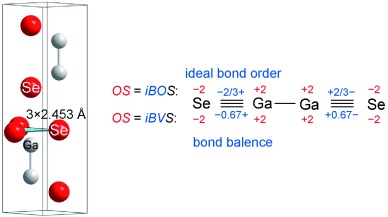
Unit cell of GaSe with one full Ga coordination shown (left, two of the three coordinated Se atoms are outside the unit cell) and the oxidation states determined from its bond graph (right). The bond order dictated by the 8−*N* rule is listed above the three connectivity lines. Below them, the bond valence is listed, calculated from the bond length determined[50] by X-ray diffraction.

When there is sufficient difference in electronegativity, bonding predictions with the generalized 8−*N* rule are precise. Consider GaSe with *VEC*_A_=9: The single electron in excess of 8 would remain on Ga, thereby forming single-bonded Ga=Ga dumbbells. That is indeed the case, and the oxidation states in GaSe are evaluated by summing bond orders in Figure 12.

**Figure 13:**
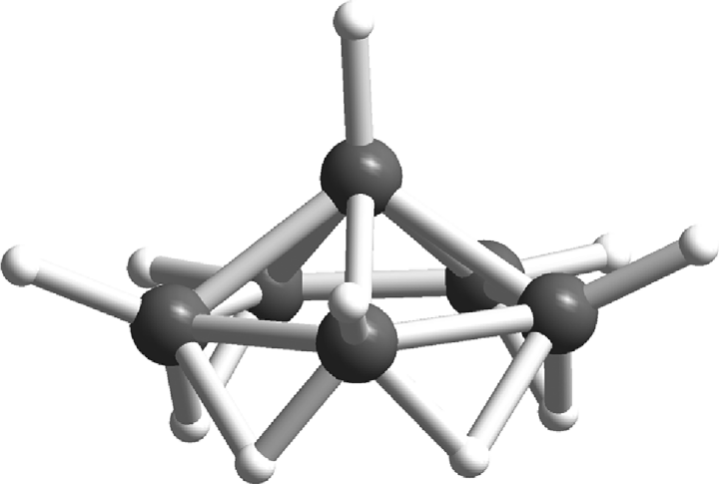
The *nido*-borane B_6_H_10_.

Boranes of general formula B_*b*_H_*h*_^*c*^ (*b*&4, *c*≤0) are sp clusters that are not electron-precise (the skeleton bonds are not single bonds). When all the B atoms are equivalent, the oxidation state can be evaluated by DIA on a repeat unit, such as BH^(1/3)−^ with 4 1/3 electrons for B_6_H_6_^2−^. The unit’s hydrogen atom obtains 2 electrons (2−*N* rule), thereby leaving 2 1/3 electrons at B, which means that *OS*_B_=+2/3 (still counting whole electrons; 2 per 3 atoms). Generalizing that *OS*_H_=−1, charge balance yields (*h*+*c*)/*b* for the average boron oxidation state in B_*b*_H_*h*_^*c*^. To resolve individual boron oxidation states, Wade–Mingos rules[51–54] are applied first: As an example, B_6_H_10_ has 14 valence-electron pairs of which 6 are in B=H single bonds, 1 always is in a radial-skeletal MO, and the remaining 7, in the tangential skeletal MOs, form a 7-vertex parent deltahedron (pentagonal bipyramid), of which one vertex is “missing” in B_6_H_10_ (a *nido*-borane; Figure 13). Its B atoms are not all equivalent. The five basal boron atoms are bonded to nine hydrogen atoms that maintain the 2−*N* rule. Summation of the bond orders with a positive sign yields an oxidation state of +2 for three of these B atoms (those in front) and +3/2 for the remaining two. The apical B atom has an oxidation state of +1 arising from one single bond to H.

## 7. What if the Compound is Metallic?

When bonding and antibonding orbitals/bands overlap in a metal, we are no longer entitled to make the ionic extrapolations as in Figure 1. However, there are simple metallic compounds with obvious oxidation states, such as the golden TiO, dark RuO_2_, or silvery ReO_3_. Some sp elements also form stoichiometric metallic compounds: Ba_3_Si_4_ obeys the Zintl concept[47] in that it forms butterfly-shaped Si_4_^6−^ anions, in which two Si atoms have two bonds and two Si atoms three bonds to each other according to the generalized 8−*N* rule, but the compound is weakly metallic.[55]

**Figure 14:**
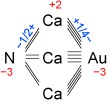
Bond graph for the metallic perovskite AuNCa_3_, in which summing bond orders (in blue) of N and Ca, obtained from 8−*N* and 8+*N* rules, respectively, yields an invalid oxidation state for Au.

Ultimately, the assignment of conducting electrons to one of the two bonded atoms has its limits. An indication of the problem is an unexpected electron configuration or an unexpected bonding pattern. The former is exemplified by the AuNCa_3_ perovskite[56] (Figure 14), where neglecting its metallic character suggests Au^3−^ anions, for which there is no support in theory. The latter may be illustrated on two platinides: red transparent Cs_2_Pt[57] and[58] black BaPt. In line with the 12−*N* rule, Cs_2_Pt contains isolated Pt^2−^ anions—a relativistic sulfide. However, BaPt does not have such anions; it has chains of Pt as if there were a deficit of electrons at the Pt atom. This means that some electrons left Pt^2−^ to make BaPt metallic, and it is this deficit that is compensated by forming Pt=Pt bonds. If these Pt=Pt bonds were single bonds, their chain would be a neutral relativistic sulfur and BaPt would be built of Ba^2+^, 2e^−^, and Pt in infinite chains. As BaPt appears formally stoichiometric, the +2 oxidation state for Ba leaves Pt with −2, which does not comply with the actual bonding.

Unsatisfactory oxidation states are also obtained when DIA is applied to ordered alloys with compositions and structures dictated largely by the size, such as LiPb or Cu_3_Au, where the 8−*N* or 12−*N* rules for the most electronegative element are not valid. If an oxidation state is needed to balance redox equations, it is best considered zero for all elements.

## 8. Nominal Oxidation States

The applications of oxidation state in chemistry are wide, and one value does not always fit all. In systematic descriptive chemistry, the oxidation state sorts out compounds of an element; in electrochemistry, it represents the electrochemically relevant compound or ion in Latimer diagrams and Frost diagrams of standard (reduction) potentials. Such purpose-oriented oxidation states that differ from those by definition may be termed nominal, here “systematic” and “electrochemical”.

**Figure 15:**
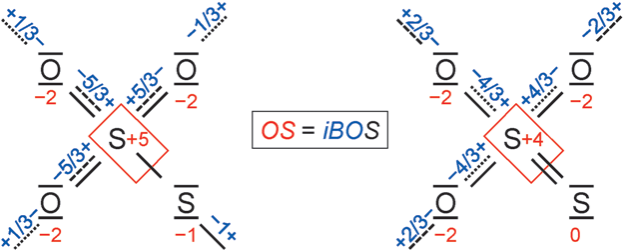
Oxidation states in thiosulfate by assignment of bonds and by summing bond orders on two limiting Lewis formulas (in which the author chose to draw bonds to unexpressed cations to avoid formal charges) with a sulfur–sulfur single bond (left), double bond (right).

An example of both is thiosulfate. Its structural properties[59] suggest that all its terminal atoms carry some of the anion charge, even if the S=S and S=O bond orders are not entirely equal. The S=S bond distance of 2.025 Å[59] is shorter than the single bond of 2.055 Å[46] in crystalline S_8_ or 2.056 Å[60] in H_2_S_2_ gas, but substantially longer than the double bond of 1.883 Å[61,62] in S_2_O or 1.889 Å[63] in S_2_. Although the single S=S bond is the closest approximation, two limiting Lewis formulas are considered in Figure 15.

The formula on the left provides oxidation state −1 at the terminal sulfur atom, reminiscent of the value in peroxides. The formula on right suggests oxidation states that at times are used for a Lewis acid–base interpretation of the synthesis reaction S + SO_3_^2−^=S_2_O_3_^2−^, making it a nonredox process. This is not necessarily an advantage, as this reaction in an aqueous environment is well-described with half-reaction standard potentials that utilize the average sulfur oxidation state +2, which represents thiosulfate in Latimer and Frost diagrams—an electrochemical oxidation state. The only route to unambiguous oxidation states for both S atoms in thiosulfate would be to resolve the S=S bond polarity, as in some textbooks: the terminal sulfur has −2, the central sulfur +6, independent of their bond order and emphasizing the similarity of the O and S ligands—a systematic oxidation state.

## 9. Non-Innocent Ligands: H_2_

Jørgensen[64,65] coined the adjective “non-innocent” for redox-active ligands that render the oxidation state of the central atom less obvious. Additional information from diffraction, spectra, or magnetic measurements is needed. Of the many examples,[66–68] the simplest non-innocent ligand is molecular H_2_.

**Figure 16:**
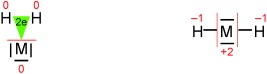
Two extremes of an H_2_ adduct with a generic metal atom M.

Complexes with molecular H_2_ resemble hapto complexes of olefins or aromatic hydrocarbons, except that H_2_ only has a *σ* bond. Despite this, H_2_ attaches to metal cations even in the gas phase, free of solvents, substrates, and intervening atoms, as elaborated in a recent review[69] by Bieske and co-workers. An intriguing ambiguity arises: with some metal ions, the atoms of the H_2_ molecule remain bonded to each other, while with others they form a dihydride. Crabtree[70] attributes this to the central transition-metal atom having both empty and filled d orbitals: The former participate in the three-center bonding MO that binds the H_2_ moiety while the latter sabotage this by back donation into the empty antibonding MO of H_2_. The two extreme outcomes are presented schematically in Figure 16.

**Figure 17:**
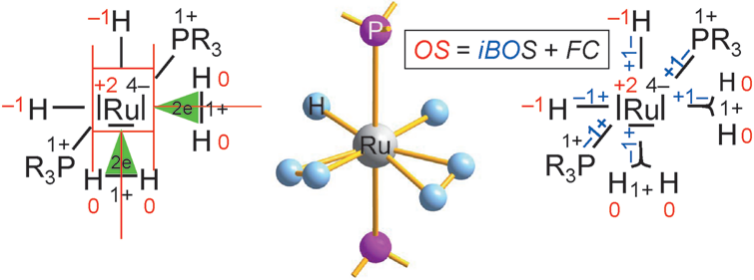
Oxidation states (in red) of hydrogen and ruthenium in [RuH_2_(η^2^-H_2_)_2_[P(C_5_H_9_)_3_]_2_],[75] obtained by assigning bonds onto the electronegative partner (left) and by summing ionized bond orders (right).

In 1984, Kubas et al.[71] synthesized the first transition-metal complex with an H_2_ ligand, the yellow [W(CO)_3_(η^2^-H_2_)[P(C_6_H_11_)_3_]_2_], by precipitating it from a solution of [W(CO)_3_[P(C_6_H_11_)_3_]_2_] in toluene with H_2_ gas. An example of the hydride formation is [Ir(CO)Cl(H)_2_[P(C_6_H_5_)_3_]_2_],[72,73] obtained from H_2_ and the square-planar [Ir(CO)Cl[P(C_6_H_5_)_3_]_2_],[74] also known as Vaska’s complex. Both types of bonded hydrogen occur in [Ru(H)_2_(η^2^-H_2_)_2_[P(C_5_H_9_)_3_]_2_],[75] the oxidation states of which are evaluated in Figure 17.

The octahedral complex in Figure 17 is stabilized by the d^6^ electronic configuration and the 18-plet on Ru. While the two *cis*-hydride anions are 2.13 Å apart, the H=H distance in the H_2_ ligand is 0.83 Å (0.09 Å longer than in H_2_ gas). Its bond order is 0.78, a little more than 2/3 for a three-center bond of equal partners, and this can be attributed to the 2−*N* rule working for the more electronegative hydrogen atom.

A review by Morris[76] of iron-group H_2_ complexes shows that the H=H bond distances vary, up to about 1.60 Å for the largest Os,[77] and also depend on the ligand *trans* to H_2_: When that ligand is an electron-rich sp atom, such as oxygen or a halogen[78] capable of strong π donation to the central atom, the H=H distance is long. When that ligand is π-acidic, such as CO, or when it is electron-poor, the H=H distance is short.[76] The distance increases with the extent of back donation from the central atom into the σ* MO of H_2_, as controlled by the metal’s size and by the *trans* ligand. By our oxidation-state definition, the back-bonding metal atom gets its electrons back because it is the main contributor to this additional metal–ligand bonding interaction, which is antibonding with respect to the H=H bond. Allen electronegativities also yield zero for the oxidation state of all such η^2^ hydrogen atoms.

## 10. Non-Innocent Ligands: Nitrosyl

Perhaps the best known nitrosyl complex is nitroprusside. While CN in [Fe(CN)_5_NO]^2−^ is easy, NO offers three alternatives for the nitrogen oxidation state: NO^+^, NO, and NO^−^. They differ in bond order: either |N]O|^+^ with *OS*_N_= +3, or |N]O|^−^ with *OS*_N_=+1 (by DIA), or the nitrogen monoxide of *OS*_N_=+2 in between.

We adopt the bond-valence approach: Single-crystal neutron diffraction of Ba[Fe(CN)_5_(NO)]⋅3 H_2_O[79] yields an NO bond length of 1.12 Å, shorter than the 1.15 Å[80] in NO gas. Considering that the 8−*N* rule for oxygen will tend to decrease the actual bond order towards two, the observed bond length suggests |N]O|^+^, hence Fe^2+^. The diamagnetism of nitroprusside confirms this: The electron configuration at Fe is low-spin d^6^ and *OS*_Fe_=+2. The octahedral field of strong splitters keeps the low-spin configuration even upon reduction to [Fe(CN)_5_NO]^3−^; it is the NO^+^ ligand that is reduced to NO not iron. A truly non-innocent ligand

Many nitrosyl complexes are not as straightforward. The MNO segment should be linear for |N]O|^+^ but bent for |N]O|^−^.[81,82] The snag is that the MNO angles vary, indicating fractional NO bond orders and problematic oxidation-state assignments.[83–85] Enemark and Feltham[86] avoided oxidation states in nitrosyl complexes altogether by adopting a [MNO]^*n*^ notation, where *n* is the number of valence electrons on the metal when the ligand is formally NO^+^.

**Figure 18:**
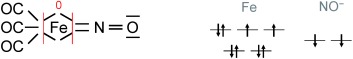
The Lewis formula of [Fe(CO)_3_(NO)]^−^ (left) illustrating the 18-plet and the oxidation state of iron by assigning bonds. Intramolecular antiferromagnetic coupling leads to diamagnetism of the anion (right).

A recent study[87] reinvestigated [Fe(CO)_3_(NO)]^−^, which seems isoelectronic with [Fe(CO)_4_]^2−^ in which iron has an oxidation state of −2—a mere replacement of CO with NO^+^. Something was not right though: the FeNO angle is linear, but the NO bond distance of 1.21 Å suggests a double bond |N]O|^−^. Spectroscopic and quantum-chemical considerations in Ref. [87] brought an explanation: The Lewis-basic N atom of the |N]O|^−^ anion donates both electron pairs as two π bonds to the Fe central atom (no σ bond), thereby linearizing the FeNO angle and validating the double bond within NO. The resulting oxidation state of 0 for iron is corroborated in Ref. [87] by the diamagnetism of the complex, caused mainly by antiferromagnetic coupling of two unpaired electrons at the tetrahedrally coordinated d^8^ iron with two unpaired electrons at the NO^−^ ligand, isoelectronic with O_2_. Ref. [87] therefore also lists these d configuration electrons in an expanded Enemark–Feltham notation: [Fe^8.2^(NO)]^10^ (8.2 was calculated). Figure 18 illustrates the stabilizing 18-plet at Fe.

## 11. Oxidation-State Tautomerism

**Figure 19:**
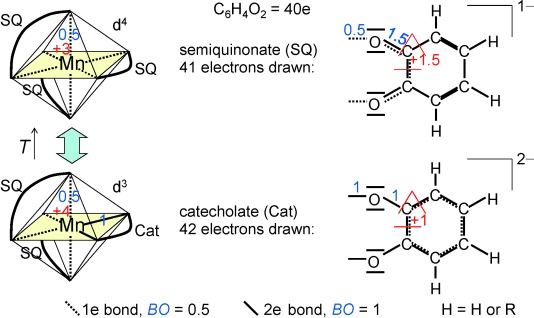
Oxidation-state tautomerism of [Mn(C_6_H_4_O_2_)_3_]. Bond orders in blue.

Oxidation-state tautomerism, also known as valence tautomerism, concerns thermally induced oxidation-state changes involving redox-active ligands and redox-prone central atoms. Manganese catecholate is an example. At low temperatures, magnetic measurements suggest [Mn(C_6_H_4_O_2_)_3_] has one catecholate and two semiquinonate ligands around a central Mn atom of oxidation state +4.[88] At high temperatures, the magnetic moment suggests reduction to high-spin Mn^3+^ upon oxidation of the catecholate to semiquinonate. Lewis formulas for the two ligand alternatives are drawn in Figure 19, where the transition is illustrated and relevant oxidation states evaluated by both algorithms. More examples have been surveyed.[89–91] An oxidation-state tautomerism among solely central atoms is exemplified in Ref. [14d].

## 12. Oxidation State and d^n^ Configuration

**Figure 20:**
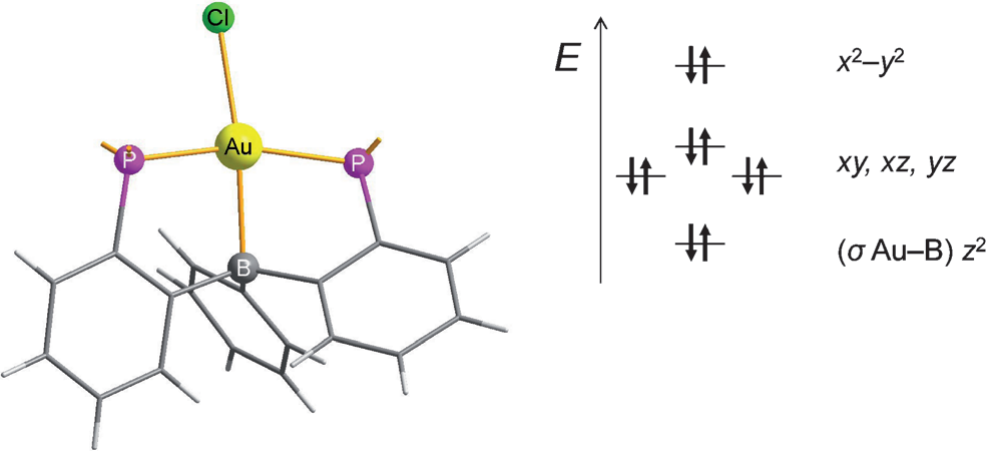
Square-planar Au in [Au[B(PC_6_H_4_)_2_(C_6_H_5_)]Cl].[92]

The configuration d^*n*^ is a central-atom descriptor for transition-metal complexes. It becomes tricky when the ligand is bonded by the more electronegative atom as a Lewis acid. One of the examples discussed in Ref. [14e] is [Au[B(PC_6_H_4_)_2_(C_6_H_5_)]Cl].[92] In this adduct, Au populates the Au=B weakly bonding MO so that the Mössbauer spectrum still sees this MO together with the rest of the d electrons at Au as d^10^, thus suggesting an oxidation state of +1 for gold, despite the square-planar coordination at Au that is typical of Au^3+^ with d^8^ (Figure 20). The square-planar Au appears because the donated Au pair became the Au=B bond itself, lost its ligand-field effect, and the coordination geometry is now controlled by the 8 electrons of Au remaining in the weakly antibonding MOs. Our generic definition also suggests +1 for gold. For this oxidation state to maintain the important formula *n*=*N*−*OS* valid for d^*n*^ at a transition-metal atom with *N* valence electrons, *n* must also include the weakly bonding pair donated by the central atom. To fulfill the equation and avoid the emerging ambiguity exemplified above by the d^10^ “spectroscopic” versus the d^8^ “ligand-field” or “magnetic” configurations, we follow Parkin[93] by noting the configuration as *n*=10 in d^*n*−2^, where “2” symbolizes a weakly bonding “donated” pair.

## 13. Choices, Estimates, and Round Offs

For intermetallic compounds, the ultimate choice of the oxidation state zero at all atoms is best if needed in redox chemistry. Usage-related choices also define the nominal oxidation states (see Section 8).

Subtler estimates and round offs are required for compounds with electrons delocalized over non-equivalent atoms, as expressed by several resonance formulas with weights in arbitrarily long decimal numbers. Without round offs of bond orders in Lewis formulas, decimal values of oxidation states would be obtained for certain bonding connectivities (to which DIA does not apply; Ref. [14] Appendix D). Examples are 1*H*-pentaazole,[14f] N_5_^+^,[14g] thiosulfate (see Section 8). Compounds with steric bonding compromises, such as S_4_N_4_ (see Section 6), are a related group. Compounds with conflicts of bond-stability rules make a similar group, illustrated in Ref. [14g] with N_2_O (DIA does not apply).

On the other hand, unambiguous and reasonable fractions of small integers are obtained for oxidation states in compounds such as dithiolate and catecholate (see Section 11) or in (car)boranes such as B_6_H_10_ (see Section 6) and B_10_C_2_H_12_,[14h] or when vicinal oxidation states are indistinguishably mixed, such as in YBaFe_2_O_5_.[14d] Reasonable fractional oxidation states appear also in ions where the charge is distributed over several equivalent atoms such as C_7_H_7_^+^, B_6_H_6_^2−^,[14i] I_3_^−^, and N_3_^−^.[14j]

Round offs are necessary for bond-valence sums after the bond-length to bond-valence conversions with Equation (2). Their decimal values are inherent to the statistical distribution of bonding compromises when the length of a given bond is compared to an average length of a selected group of reference bonds. In addition, an empirical function is used for the bond-length to bond-order conversion.

## 14. Outlook on Computational Approaches

**Figure 21:**
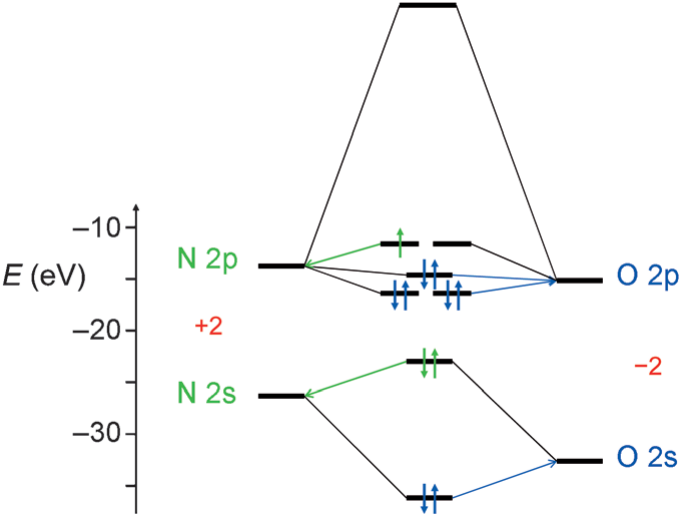
Oxidation states in nitrogen monoxide by assigning the MO’s electrons to the energetically closest AOs in a MO set deduced from the orbital energies estimated with an extended-Hückel program.[94]

The generic definition in Ref. [14] states: “*The oxidation state of a bonded atom equals its charge after ionic approximation*”. Only heteronuclear bonds are extrapolated to be ionic, and the atom to become negative is the one that contributes more to the bonding MO. The heuristic MO diagram in Figure 1 does suggest that quantum-chemical calculations might be used to evaluate oxidation states. As discussed in Appendix C of Ref. [14], this carries an inherent degree of ambiguity because of the variety of computational methods available and of the basis-set data to choose from. Within this limitation, a possible MO approach might use a generalization stating that an atom reversibly contributing more to a given MO or MO* of a heteronuclear bond keeps that MO’s electrons,[14k] with a built-in condition that homonuclear bonds are split evenly. This is illustrated with nitrogen monoxide in Figure 21. Somewhat obscured by the sp interaction, we see that a MO is closer in energy to one of its two contributing AOs. That AO then receives the MO’s electrons upon ionic approximation. When repeated over all the MOs, the expected oxidation states are obtained (Figure 21).

**Figure 22:**
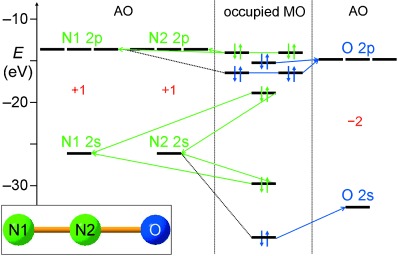
Oxidation states in N_2_O by assigning the MO’s electrons to the energetically closest AO in a MO set from an extended-Hückel program[94] calculation. Four unoccupied MOs are omitted.

A molecule with a homonuclear bond, N_2_O, is treated similarly in Figure 22. During such a “manual” approach, one has to identify atoms that are actually or predominantly bonded together by each particular MO. Although merely illustrative of a conceptual suggestion, the oxidation-state approach in Figure 22 circumvents the dilemma encountered in Ref. [14f] over two alternative Lewis formulas of N_2_O.

## 15. A Summary of the Algorithms

A simplified approach to the ionic approximation and oxidation states identifies the negative atom by comparing Allen electronegativities with the exception of the more electronegative atom being bonded as a Lewis acid. This approach comes in three algorithms for three different types of chemical formulas (summary formula, Lewis formula, bond graph) covering molecules, ions, and 1D (chains), 2D (planes), or 3D infinite networks of solids. An overview of the inputs and validity is given in Table 2.

**Table 2:** Overview of the three oxidation-state algorithms based on ionic approximation according to electronegativity.

Algorithm	Valid for	Input	Performed on
direct ionic approximation	homoleptic binaries of sp terminal atoms	summary formula, total number of valence electrons	individual atom symbols of the summary formula
			
assigning bonds	finite molecules or ions^[a]^	Lewis formula of the above, showing all bond pairs and lone pairs, and, if present, all fractional bond pairs and fractional lone pairs	Lewis formula
			
summing bond orders	finite molecules or ions^[a]^	Lewis formula with all of the above, and formal charges for all atoms	Lewis formula
		
	solids with 1D, 2D,^[b]^ or 3D infinite networks	bond graph with all bonding connectivities marked as lines, bond order (bond valence) for all connectivity lines	bond graph

[a] The simplifying use of electronegativity as a criterion for the ionic approximation (Figure 1 versus Figure 2) carries an exception: In reversible adducts of a Lewis-acidic atom with an electronegativity higher than its Lewis-basic counterpart, the base keeps the donated pair. [b] In 1D and 2D networks, possible formal charges of atoms at molecule-like edges or faces need to be identified on the bond graph (see example in Figure 8).

## 16. Conclusion

The suggested oxidation-state definition justifies both IUPAC algorithms in Ref. [3], while removing exceptions and including some defiant cases, such as those of ligand acceptor atoms with an electronegativity higher than the donor. Being based on chemical bonding, our definition does not replace the algorithms needed early on in the chemistry curriculum. It might be helpful at a higher level.
